# HIV Prevalence and Undiagnosed Infection among a Community Sample of Gay and Bisexual Men in Scotland, 2005–2011: Implications for HIV Testing Policy and Prevention

**DOI:** 10.1371/journal.pone.0090805

**Published:** 2014-03-12

**Authors:** Lesley A. Wallace, Jessica Li, Lisa M. McDaid

**Affiliations:** 1 Health Protection Scotland, National Services Scotland, Glasgow, Scotland, United Kingdom; 2 Section of Public Health, University of Sheffield, Sheffield, England, United Kingdom; 3 MRC/CSO Social and Public Health Sciences Unit, University of Glasgow, Glasgow, Scotland, United Kingdom; Public Health Agency of Barcelona, Spain

## Abstract

**Objective:**

To examine HIV prevalence, HIV testing behaviour, undiagnosed infection and risk factors for HIV positivity among a community sample of gay men in Scotland.

**Methods:**

Cross-sectional survey of gay and bisexual men attending commercial gay venues in Glasgow and Edinburgh, Scotland with voluntary anonymous HIV testing of oral fluid samples in 2011. A response rate of 65.2% was achieved (1515 participants).

**Results:**

HIV prevalence (4.8%, 95% confidence interval, CI 3.8% to 6.2%) remained stable compared to previous survey years (2005 and 2008) and the proportion of undiagnosed infection among HIV-positive men (25.4%) remained similar to that recorded in 2008. Half of the participants who provided an oral fluid sample stated that they had had an HIV test in the previous 12 months; this proportion is significantly higher when compared to previous study years (50.7% versus 33.8% in 2005, p<0.001). Older age (>25 years) was associated with HIV positivity (1.8% in those <25 versus 6.4% in older ages group) as was a sexually transmitted infection (STI) diagnosis within the previous 12 months (adjusted odds ratio 2.13, 95% CI 1.09–4.14). There was no significant association between age and having an STI or age and any of the sexual behaviours recorded.

**Conclusion:**

HIV transmission continues to occur among gay and bisexual men in Scotland. Despite evidence of recent testing within the previous six months, suggesting a willingness to test, the current opt-out policy may have reached its limit with regards to maximising HIV test uptake. Novel strategies are required to improve regular testing opportunities and more frequent testing as there are implications for the use of other biomedical HIV interventions.

## Introduction

Transmission of HIV continues to occur among men who have sex with men (MSM) in Scotland and the rest of the UK. There have been annual increases in the number of reported diagnoses in the past ten years; these reports represent a combination of both recently acquired and longstanding HIV infection [1]. This is partly due to increased testing uptake, most probably as a result of the change to opt-out HIV testing when an HIV test is offered in the context of routine, recommended sexual health testing to those attending specialist services, for example, genitourinary medicine (GUM) clinics – a policy implemented as part of the Scottish Government's Sexual Health Strategy and Action Plan in the early part of the last decade to normalise HIV testing [2]. Between 2003, when opt-out testing began, and 2008 an 83% increase (to 3868 tests) was observed [3]. There was no change in the HIV prevalence [3], [4]. Thus, increased test uptake does not fully explain the sustained epidemic in this group, and there is evidence of new transmissions occurring [1]–[5].

The continuing transmission of HIV is a public health concern. A study in Scotland among those undergoing repeat HIV testing estimated HIV incidence among MSM (15 per 1000 person years) was three to four fold higher than other populations at risk of, and tested for, HIV: this has remained unchanged since the late 1980s [6]. Recent data suggest that one in five HIV infected MSM remain undiagnosed and it is estimated that this population accounts for around half of all new HIV transmissions [1], [7], [8]. In 2008, we reported that the sustained increase in HIV testing among MSM in Scotland was accompanied by a (non-significant) decrease in undiagnosed infection (from 40% to 25% of those testing HIV-positive) [9]. In this paper we examine HIV prevalence, HIV testing and the risk factors associated with HIV positivity among a community sample of gay and bisexual men in Scotland in 2011 and explore whether there has been further change since 2008.

## Methods

Using a form of time and location sampling [9], the MRC Gay Men's Sexual Health Survey collected anonymous, self-complete questionnaires and (Orasure) oral fluid specimens from representative samples of MSM in 17 gay commercial venues (15 bars and two saunas) in Glasgow and Edinburgh in May 2011. A team of temporary fieldworkers distributed and collected questionnaires in bars at two different time points in the early (19:00–21:00), and late evening (21:00–23:00) over the two week survey period. No bar was visited twice in the same evening and at the end of the survey period each bar had been visited at both time points on each day of the week. Each sauna was visited six times during the survey; for a two hour period (17:00–19:00) on Thursdays and between 14:00–17:00 on Saturdays and Sundays. Fieldworkers were trained to ask men if they had already completed the survey to ensure no duplicate reporting. When approached more than once in the venues men were asked to acknowledge that they had already completed the questionnaire and this was recorded on the response rate forms. Ethical approval was granted by University of Glasgow College of Social Sciences Ethics Committee. Copies of the questionnaires are available on the study website http://gaymensurvey.sphsu.mrc.ac.uk/data-sharing.

Questionnaires included questions related to demographics, HIV testing, self-reported HIV status, STI experience, and sexual risk behaviours (number of sexual partners, anal sex partners, and unprotected anal intercourse [UAI] partners in the previous 12 months). Oral fluid specimens were analysed at the West of Scotland Specialist Virology Centre. These were tested for anti-HIV using the Vironostika HIV Uni-Form II Ag/Ab enzyme immunoassay. Positive samples were re-tested and, if repeatedly reactive, were confirmed using western blot. Chi-square and Fisher's Exact tests were used for bivariate comparisons and multivariate logistic regression models, adjusting for age, qualification level, employment status and frequency of gay scene use, which differed between the surveys, were used to estimate odds ratios (ORs) and 95% confidence intervals.

1515 men participated in the survey (65.2% response rate (RR)): 848 in Glasgow (66.4% RR) and 667 in Edinburgh (63.7% RR). 1218 men provided oral fluid specimens (80.4% of men surveyed; 52.4% RR): 687 in Glasgow (81.0% of men surveyed; 53.8% RR) and 531 in Edinburgh (79.7% of men surveyed; 50.6% RR). One man from Edinburgh provided an oral fluid specimen but did not complete a questionnaire and was therefore excluded from the analyses.

## Results

### Sample characteristics

The 297 men (19.6%) who completed questionnaires but did not provide oral fluid specimens were compared with the 1217 who provided specimens. Men who provided specimens were more likely to be younger, report UAI with 2+ partners, UAI with casual partners, and UAI with partners of unknown or discordant HIV status. When controlling for the significant factors in multivariate analysis, men aged 36–45 compared to those aged 16–25 were less likely to have provided a specimen (adjusted odds ratio, AOR 0.68, 95% confidence interval, CI, 0.48 to 0.97) and men with casual UAI partners (AOR 1.60, 95% CI 1.02 to 2.52) were more likely to have provided an oral fluid specimen. There were no differences in HIV testing or self-reported HIV status between the two groups.

### HIV Prevalence

Of the 1217 men who provided an oral fluid specimen, 59 were HIV-positive (4.8%, 95% CI: 3.8% to 6.2%) (Table 1). HIV prevalence was 4.4% in Glasgow (95% CI: 3.1% to 6.2%) and 5.5% in Edinburgh (95% CI: 3.8% to 7.7%). The factors associated with having a positive test result are shown in Table 1. At the bivariate level, age and having an STI in the previous 12 months were significantly associated with a positive test result (Table 1). Prevalence was significantly higher among men older than 25 (with a mean age of 37 for positive men and 32 for negative men) and was lowest among men who had never been tested (0.5%). After adjusting for age (data not shown), having a positive test result was also significantly higher among men who reported UAI with two or more partners in the previous 12 months (AOR 1.98, 95% CI 1.03 to 3.80) and having a sexually transmitted infection (STI) in the previous 12 months (AOR 2.57, 95% CI 1.37 to 4.80). When all sexual behaviours were controlled for, in addition to age, in the multivariate model (data not shown), the odds remained significantly higher for men aged 26–35 (AOR 2.74, 95% CI 1.11 to 6.78), 36–45 (AOR 6.51 95% CI 2.72 to 15.60), and 46+ (AOR 2.98, 95% CI 1.04 to 8.52) compared to men aged 16–25, and those who had experienced an STI in the previous 12 months (AOR 2.13, 95% CI 1.09 to 4.14). There were no significant interactions found between age and STI experience or age and any of the sexual behaviours.

**Table 1:** HIV status by demographic characteristics and behavioural risk factors of MSM who answered the questionnaire and provided an oral fluid specimen (N = 1217), 2011.

	HIV status		Odds ratio (OR) for positive test result (Unadjusted)	
	HIV+/Total	(%)	OR	95% CI
**Survey location**				
Edinburgh	29/530	5.5	1	
Glasgow	30/687	4.4	0.79	(0.47–1.33)
**Survey venue**				
Bar	57/1166	4.9	1	
Sauna	2/51	3.9	0.79	(0.19–3.35)
**Age**				
16–25	7/385	1.8	1	
26–35	18/402	4.5	2.53	(1.05–6.13)
36–45	26/252	10.3	6.21	(2.65–14.55)
46+	8/164	4.9	2.77	(0.99–7.77)
**Area of residence**				
Rest of Scotland	9/189	4.8	1	
Edinburgh	23/390	5.9	1.25	(0.57–2.76)
Glasgow	20/513	3.9	0.81	(0.36–1.82)
Rest of UK	4/60	6.7	1.43	(0.42–4.82)
**Educational Qualifications**				
Secondary	10/179	5.6	1	
Further/vocational	21/425	4.9	0.88	(0.41–1.91)
Degree/postgraduate	25/526	4.8	0.84	(0.40–1.79)
**Employment**				
Not employed	50/950	5.3	1	
Employed	9/261	3.4	0.64	(0.31–1.33)
**Frequency of gay scene use**				
Once a month or less	22/353	6.2	1	
2–3 times a month	14/302	4.6	0.73	(0.37–1.46)
Once or more a week	23/551	4.2	0.66	(0.36–1.20)
**HIV testing history**				
In past year	38/610	6.2	1	
Between 1–5 years ago	13/305	4.3	0.67	(0.35–1.28)
Over 5 years ago	7/72	9.7	1.62	(0.70–3.78)
Never tested	1/215	0.5	0.07	(0.01–0.52)
**Number of sexual partners**				
<10	37/899	4.1	1	
10+	19/293	6.5	1.62	(0.91–2.86)
**Number of anal sex partners**				
<10	45/1057	4.3	1	
10+	10/130	7.7	1.87	(0.92–3.82)
**UAI with 2 or more partners**				
0–1 partner	45/1026	4.4	1	
2 or more partners	13/173	7.5	1.77	(0.94–3.36)
**UAI with casual partners**				
No	43/894	4.8	1	
Yes	15/305	4.9	1.02	(0.56–1.87)
**UAI with partners of unknown or discordant status**				
No	38/907	4.2	1	
Yes	21/310	6.8	1.66	(0.96–2.88)
**Had STI other than HIV in previous 12 months**				
No	44/1039	4.2	1	
Yes	15/151	9.9	2.49	(1.35–4.60)

### HIV testing and undiagnosed Infection

Half of the sample of men who provided an oral fluid specimen (610 of 1202, 50.7%) said that they had undergone an HIV test in the previous 12 months. Of the 59 men who tested positive, 44 (74.6%) reported that their last HIV test result was positive, 14 (23.7%) reported that it was negative, and one did not report his test result (but he perceived himself to be HIV-negative) indicating that 15 men (25.4%) were undiagnosed at the time of the survey. Of these, 13 perceived themselves to be HIV-negative and two reported that they did now know their HIV status; none perceived themselves to be HIV-positive. Furthermore, four reported having tested (HIV-negative) in the previous six months and three reported testing between six to 12 months previously. In comparison, 26 of 44 (59.1%) diagnosed HIV-positive men had tested in the previous six months and a further five (11.4%) had tested between six to twelve months previously.

The testing behaviour of diagnosed and undiagnosed HIV-positive men was examined by comparing their reasons for testing and the locations at which they had tested in the previous 12 months (Table 2). Note the small numbers in each group suggest caution in interpreting these results. Fourteen diagnosed men reporting having an STI other than HIV in the previous 12 months compared with only one undiagnosed man (Table 2). Among men tested for HIV and/or other STIs in the previous 12 months, most reported testing at specialist sexual health, including gay-specific, services (23/36 diagnosed men, 6/8 undiagnosed men and 458/594 HIV-negative men) (data not shown). Venue and home testing were not used by the HIV diagnosed and undiagnosed men but were reported by a small number of HIV-negative men (n = 34). Testing in the primary care setting was reported by 105 (18.1%) HIV-negative men, but only two diagnosed HIV-positive men and two undiagnosed men.

**Table 2:** HIV infection status and sexual health testing behaviour of MSM who answered the questionnaire and provided an oral fluid specimen (N = 1217), 2011.

	Diagnosed (N = 44), n (%)	Undiagnosed (N = 15), n (%)	Negative (N = 1158), n (%)	p value
**HIV testing history**				
In previous 6 months	26 (59.1)	4 (26.7)	371 (32.5)	0.001*
Between 6–12 months ago	5 (11.4)	3 (20.0)	201 (17.6)	
Between 1–5 years ago	7 (15.9)	6 (40.0)	292 (25.5)	
Over 5 years ago	6 (13.6)	1 (6.7)	65 (5.7)	
Never had a test	0 (0.0)	1 (6.7)	214 (18.7)	
**Reason for HIV test**				
Regular test or check up	29 (74.4)	9 (64.3)	570 (65.1)	0.210*
New or other partner risk	1 (2.6)	1 (7.1)	106 (12.1)	
Risk event	7 (17.9)	4 (28.6)	188 (21.5)	
Other	2 (5.1)	0 (0.0)	12 (1.4)	
**Had STI other than HIV in previous 12 months**				
No	30 (68.2)	14 (93.3)	995 (88.0)	<0.001
Yes	14 (31.8)	1 (6.7)	136 (12.0)	
**STI (other than HIV) testing history**				
In last 6 months	29 (65.9)	5 (38.5)	370 (33.0)	<0.001*
Between 6–12 months ago	5 (11.4)	4 (30.8)	216 (19.3)	
Over 12 months ago	10 (22.7)	3 (23.1)	327 (29.1)	
Never had an STI test	0 (0.0)	1 (7.7)	209 (18.6)	

*Fisher's Exact test.

### Comparison of HIV prevalence, testing and undiagnosed infection between 2005, 2008 and 2011

The HIV prevalence rate is similar across the three surveys and the proportion of HIV-positive men who were undiagnosed remained unchanged between 2008 and 2011. HIV testing in the previous 12 months increased significantly between 2005 and 2011; half of the sample reported having tested in the previous 12 months in 2011 compared with one third in 2005 (Table 3). However, when directly comparing 2008 and 2011, the increase over the last three years was not significant (AOR = 1.16, 95% CI 0.99–1.37, p = 0.07). We also examined the trend in self-reported sexual risk behaviours between 2005 and 2011 (Table 3). There was a significant increase in UAI with casual partners from 19.8% in 2005 to 25.4% in 2011. There was also a significant change in self-reported STIs over time: following a decrease between 2005 and 2008, the proportion reporting this returned to the 2005 level in 2011 (Table 3). When directly comparing 2008 and 2011 and adjusting for age, qualification level, employment status and frequency of gay scene use, which differed between the surveys, the increase in self-reported STIs between the two years was significant (AOR = 1.49, 95% CI 1.15–1.94, p = 0.003).

**Table 3:** HIV prevalence, self-reported HIV status, HIV and sexually transmitted infection testing behaviour among MSM providing an oral fluid specimen across the survey years 2005, 2008 and 2011.

	2005 (n = 1350)		2008 (n = 1277)		2011 (n = 1217)		
	n	%	n	%	n	%	p Value
**HIV status (oral fluid test result)**							
Negative	1284	95.5	1216	95.5	1158	95.2	
Positive	60	4.5	57	4.5	59	4.8	
Adjusted odds of having a positive test result (95% CI)*	1		1.03 (0.70–1.51)		1.16 (0.79–1.71)		0.713
**Undiagnosed HIV (among men tested HIV positive)**							
Diagnosed	35	58.3	42	73.7	44	74.6	
Undiagnosed	25	41.7	15	26.3	15	25.4	
Adjusted odds of having a positive test result (95% CI)*	1		0.59 (0.25–1.39)		0.53 (0.22–1.24)		0.285
**Had HIV test in previous 12 months**							
No	824	66.2	648	52.8	592	49.3	
Yes	421	33.8	580	47.2	610	50.7	
Adjusted odds of having a positive test result (95% CI)*	1		1.83 (1.55–2.17)		2.12 (1.79–2.51)		<0.001
**Had STI other than HIV in previous 12 months**							
No	1137	87.5	1137	91.1	1039	87.3	
Yes	163	12.5	111	8.9	151	12.7	
Adjusted odds of having a positive test result (95% CI)*	1		0.71 (0.55–0.92)		1.08 (0.84–1.38)		0.005
**Number of unprotected anal intercourse (UAI) partners**							
0/1 partner	1141	85.7	1076	87.6	1026	85.6	
2+ partners	190	14.3	153	12.4	173	14.4	
Adjusted odds of having a positive test result (95% CI)*	1		0.93 (0.73–1.17)		1.12 (0.89–1.41)		0.275
**UAI with casual partners**							
No	1073	80.2	981	79.8	894	74.6	
Yes	265	19.8	248	20.2	305	25.4	
Adjusted odds of having a positive test result (95% CI)*	1		1.12 (0.92–1.37)		1.57 (1.29–1.90)		<0.001
**UAI with unknown or discordant partners**							
No	989	73.6	934	76.0	889	74.1	
Yes	349	26.1	295	24.0	310	25.9	
Adjusted odds of having a positive test result (95% CI)*	1		0.95 (0.79–1.15)		1.06 (0.88–1.28)		0.512

*Adjusted for age, qualification level, employment status and frequency of gay scene use, which differed between the surveys.

## Discussion

HIV testing is considered to be one of the most important measures in reducing HIV transmission among MSM and testing uptake in Scotland increased in the early part of the last decade, largely as a result of the introduction of opt-out testing [2]–[4]. In 2008, there was a concurrent decrease in undiagnosed HIV amongst our community-based survey participants [9]. However, the data presented here indicate that HIV prevalence has remained stable between 2005 and 2011, the level of undiagnosed HIV infection in MSM in 2011 remains the same as that recorded in 2008 (at one quarter of the positive cohort in this study), and there has been only a marginal further increase in HIV testing. Whilst there is evidence from national surveillance data that indicates new transmissions occurred during this time, national data also indicate little change in the prevalence [3]. In a recently published modelling study using data on MSM in England and Wales over the ten year period 2001 to 2010, there was no decrease in incidence despite an increase in HIV testing rates and high treatment coverage [10]. This study is not designed to measure incidence due to a number of limitations in its design; even a crude measure, using the self-reported HIV testing interval which is not reported by all study participants, estimates a level higher than that described in the Scottish study by McDonald et al which utilised locally collected testing data applicable to this population [6]. Notwithstanding the need for measuring incidence in this population, these results, along with the previous study results, provide valuable insights into the HIV testing and infection status of our MSM population over time. The undiagnosed proportion in our study is similar to the estimate for MSM at the UK level and presents further challenges for the prevention of HIV infection in this group [1], [11]. If the epidemic is being sustained through transmission from the undiagnosed fraction, as is commonly believed, what further actions are required to increase testing and ultimately reduce this?

Just over half of the sample reported having an HIV test in the previous 12 months, regardless of HIV status. Unsurprisingly, HIV testing within the previous six months was highest among men living with diagnosed HIV (reported by 59.1% of men compared with 32.5% or 26.7% of men who were HIV-negative or undiagnosed, respectively) as this likely reflects visits for HIV specialist care where blood samples are taken for viral load measurement in those who knew their HIV diagnoses, or for repeat testing to confirm a recent diagnosis. However, this cannot be established from our data as men were not asked their date of diagnosis or their reason for their most recent HIV test. Among men with undiagnosed HIV, it is interesting to note that their reason for testing was as part of a routine check up or following a risk event. This suggests a willingness to test and a level of awareness that offers the opportunity for targeted HIV prevention including the promotion of frequent HIV testing among those at highest risk.

Our results suggest that more must be done to increase the proportion of ‘at risk’ men testing as frequently and regularly as guidelines recommend. In the UK, the BHIVA/BASHH guideline recommends at least annual testing or more frequently if there is ongoing high risk exposure, or clinical symptoms suggestive of seroconversion in MSM and repeat testing among those who test negative but where an exposure may have occurred during the window period [12]. This is a similar approach recommended for those at risk by the CDC [13], however, these guidelines also recommend an opt-out testing strategy for all patients in all health care settings no matter their presenting condition. This contrasts with the UK policy which recommends expanded testing according to local prevalence. HIV test uptake is good among those attending GUM services where only 9% are known to decline an HIV test [14]. The challenge is how to further improve testing opportunities and to encourage more frequent testing.

While our data indicate that most men in the survey are using conventional HIV testing venues, these may not satisfy the needs of all those at risk of infection who wish a test. In a recent survey to examine barriers to HIV testing, over a ten year period to 2010, among MSM in Glasgow, it was apparent that while HIV testing has been partially normalised during this time, both behavioural (for example, fear of a positive test) and structural barriers (such as clinic opening times and testing waiting times) remained [15]. It is essential that these barriers to testing be addressed if HIV test normalisation is to be fully accepted. One strategy could be to increase opportunities for testing in convenient locations, such as through outreach (including saunas if feasible and acceptable to staff and clients) and community-based clinics using rapid HIV tests. These strategies (notwithstanding some concerns about confidentiality and availability of post-test counselling) have been successful in reducing barriers to testing, targeting populations at risk, diagnosing HIV in those who were unaware of their infection, and were acceptable to clients and staff [16]–[20]. Furthermore, the use of home testing kits for HIV (which will become legally available in the UK in April 2014) may be explored as a method to increase, and help to normalise, HIV testing.

In the multivariate analysis, the most significant finding was that men with a self-reported STI diagnosis in the past 12 months were twice as likely to be HIV-positive. It is also worthy of note that when comparing diagnosed, undiagnosed and HIV-negative men, it was men living with diagnosed HIV who reported the highest level of STI diagnoses despite similar levels of STI testing among diagnosed and undiagnosed men. It is, however, unknown whether their STI test was prior to, after, or concurrent with the HIV diagnosis and it is not possible to determine with which one or more of the other STIs men were diagnosed. Whilst the likelihood of HIV acquisition (and transmission) is increased in the presence of infection with other major acute STIs, particularly rectal gonorrhoea [21], [22] and levels of STI and HIV co-infection in this population remain a concern [23], [24], an increase in UAI with casual partners was also apparent in 2011; the first such change in sexual risk behaviour evident in almost a decade [25], [26]. In addition, we have recently shown that STI and HIV testing are strongly associated, and as such, sexual health screening presents an opportunity for HIV prevention with HIV-negative and HIV-positive men [27]. STI management, including increasing the frequency of sexual health checkups, possibly via a recall system (similar to that used in screening programmes and other areas of the health service), with rapid treatment, HIV testing for HIV-negative men, and continued HIV prevention and safer sex advice should be an essential component of the HIV prevention toolkit.

There are, however, some limitations to our study. This is a community based sample, which will not be generalisable to all MSM living in Scotland, although it is set in the two largest cities where the majority of new HIV infections are reported. The use of self-completed questionnaires may result in some recall and reporting biases, but the anonymous nature of the survey should attempt to limit these. Note also that the anonymous nature of the survey means that we cannot identify and exclude men who may have completed the questionnaire more than once in any given year; the number is thought to be very small given the short timeframe of data collection and the training of fieldworkers to avoid this. In addition, it is not possible, due to the anonymity inherent to the cross-sectional nature of the study, to identify men who have participated in more than one of the triennial surveys and thus, determine the seroconversion rate. There are a small number of HIV-positive men and of these, a small number who were undiagnosed at the time of the survey, suggesting caution in the interpretation of these results. It should be noted that while there is an association between HIV serostatus and behaviour, this is not causal as one may be a consequence of the other in a bidirectional manner. Notwithstanding these caveats, these results are comparable in response rates and the proportion providing oral fluid specimens to our previous surveys [9], [28]. Although younger men (aged less than 25) and men reporting UAI with casual partners were more likely to provide oral fluid specimens, there were no differences in HIV testing behaviours or in self-reported HIV status.

It is recognised that a combination prevention approach is likely to be the key to reducing the burden of HIV infection among MSM [29], [30]. HIV testing is an important component of this [31] and the stabilisation in rates of HIV testing and undiagnosed infection in our sample between 2008 and 2011 suggest the opt out policy, implemented in the early part of the first decade of this century, may have reached its limit in terms of maximising the uptake of HIV testing as a routine test in this population. Further efforts to improve testing opportunities and encourage more frequent testing are required and should evaluate greater use of community-based and rapid testing initiatives. New biomedical HIV prevention strategies, including treatment as prevention (TasP), present new opportunities but also require frequent and regular testing if they are to be effective [29], [30]. Our results suggest there is indeed more to be done to increase the frequency of HIV testing to the extent required for TasP to be effective in our prevention efforts.
